# Deuterium oxide as a contrast medium for real-time MRI-guided endovascular neurointervention

**DOI:** 10.7150/thno.55953

**Published:** 2021-04-15

**Authors:** Lin Chen, Jing Liu, Chengyan Chu, Zheng Han, Nirhbay Yadav, Jiadi Xu, Renyuan Bai, Verena Staedtke, Monica Pearl, Piotr Walczak, Peter van Zijl, Miroslaw Janowski, Guanshu Liu

**Affiliations:** 1 Department of Electronic Science, Fujian Provincial Key Laboratory of Plasma and Magnetic Resonance, School of Electronic Science and Engineering, National Model Microelectronics College, Xiamen University, Xiamen, Fujian, China.; 2 Russell H. Morgan Department of Radiology and Radiological Science, Johns Hopkins University School of Medicine, Baltimore, MD, USA.; 3 The First Affiliated Hospital of Jinan University, Guangzhou, Guangdong, China.; 4 Department of Diagnostic Radiology and Nuclear Medicine, University of Maryland, Baltimore, MD, USA.; 5 F.M. Kirby Research Center for Functional Brain Imaging, Kennedy Krieger Institute, Baltimore, MD, USA.; 6 Department of Neurosurgery, Johns Hopkins University, Baltimore, MD, USA.; 7 Department of Neurology, Johns Hopkins University, Baltimore, MD, USA.

**Keywords:** deuterium oxide, MRI contrast medium, intra-arterial hyperosmotic blood brain barrier (BBB) opening, endovascular neurointervention, MRI guidance.

## Abstract

**Rationale:** Endovascular intervention plays an important role in the treatment of various diseases, in which MRI-guidance can potentially improve precision. However, the clinical applications of currently available contrast media, including Gadolinium-based contrast agents and superparamagnetic iron oxide particles (SPIO), are hindered by safety concerns. In the present study, we sought to develop D_2_O as a novel contrast agent for guiding endovascular neurointervention.

**Methods:** Animal studies were approved by institutional ACUC and conducted using an 11.7 T Bruker Biospec system and a 3T Siemens Trio clinical scanner for rodent and canine imaging, respectively. The locally selective blood brain barrier opening (BBBO) in rat brains was obtained by intraarterial (IA) injection of mannitol. The dynamic T_2w_* EPI MRI sequence was used to study the trans-catheter perfusion territory by IA administered SPIO before mannitol administration, whereas a dynamic T_1w_ FLASH sequence was used to acquire Gd contrast-enhanced MRI for assessing BBBO after injection of mannitol. The contrast generated by D_2_O assessed by either EPI or FLASH methods was compared with the corresponding results assessed by SPIO or Gd. The utility of D_2_O MRI was also demonstrated to guide drug delivery to glioma in a mouse model. Finally, the clinical utility of D_2_O-MRI was demonstrated in a canine model.

**Results:** Our study has shown that the contrast generated by D_2_O can be used to precisely delineate trans-catheter perfusion territory in both small and large animals. The perfusion territories determined by D_2_O-MRI show moderate correlation with those by SPIO-MRI (Spearman coefficient r = 0.5234, P < 0.001). Moreover, our results show that the perfusion territory determined by D_2_O-MRI can successfully predict the areas with BBBO after mannitol treatment similar to that assessed by Gd-MRI (Spearman coefficient r = 0.6923, P < 0.001). Using D_2_O-MRI as imaging guidance, the optimal infusion rate in the mouse brain was determined to be 150 µL/min to maximize the delivery efficacy to the tumor without serious off-target delivery to the brain parenchyma. The enhanced drug delivery of antibodies to the brain tumor was confirmed by fluorescence imaging.

**Conclusion:** Our study demonstrated that D_2_O can be used as a negative MRI contrast medium to guide endovascular neurointervention. The established D_2_O -MRI method is safe and quantitative, without the concern of contrast accumulation. These qualities make it an attempting approach for a variety of endovascular procedures.

## Introduction

Intra-arterial (IA) drug delivery has recently become an important branch of endovascular neurointervention for treating a variety of central nervous system (CNS) diseases 1. Direct endovascular infusion through a catheter placed in the carotid or vertebral arteries can deliver drugs at high, virtually undiluted, local concentration to the targeted sites 2. Moreover, IA infusion can be combined with blood-brain barrier (BBB) opening strategies to further improve the drug delivery efficiency in the regions with intact BBB. For example, Charest *et al.* reported that, compared with IA infusion alone, transiently opening the BBB with mannitol could result in an overall 2-fold higher carboplatin accumulation in experimental brain tumors (an 80-time increase in the nucleus of tumor cells) 3.

Real-time imaging guidance is crucial for accurately visualizing target vessels during catheter advancement and prediction of the affected tissue region, hence warranting the success of endovascular neurointervention. While fluoroscopy is still the standard of practice, increasing interests have been focused on MRI-based guidance because MRI has superb soft-tissue contrast and no ionizing radiation, and can provide morphological and functional assessments that are critical for treatment planning and monitoring. Recent developments in hardware and software have provided modern MRI with a satisfactory spatial and temporal resolution, thereby making MRI a better interactive tool for guiding endovascular intervention in real-time. Very often, contrast media, including Gadolinium-based contrast agents (GBCA) 4, superparamagnetic iron oxide particles (SPIO) 5 and carbon dioxide 6, are used to improve the conspicuity and thereby ensure the accuracy of intervention. However, the widespread clinical use of these agents is hampered by safety concerns.

In the present study, we sought to develop deuterium oxide (D_2_O) as a novel contrast agent to guide endovascular neurointervention. D_2_O is a stable isotopic form of water with similar physical and chemical properties as regular water. Previous studies demonstrated that D_2_O can be used as a diffusible tracer to measure blood flow and tissue perfusion 7. While traditionally D_2_O is only visible using ^2^H-tuned MRI hardware, the negative contrast of D_2_O in the ^1^H MRI caused by the H_2_O-D_2_O replacement effect (also known as proton replace effect) has been used to noninvasively study water dynamics in Wood Xylem 8 and precisely measure the relaxation times of fat in excised breast tissue samples^9^. Recently, Wang et al. also showed the utility of D_2_O as a negative contrast medium in ^1^H-MRI for assessing cerebral perfusion in rat brains 10. Inspired by these studies, we rationalize that the D_2_O-based contrast can be utilized to track the spatial distribution of endovascularly infused drug solutions. Transcatheter infusion of D_2_O, even in a small quantity, is expected to generate sufficiently high contrast as a high local concentration of D_2_O is present in the arteries distal to the catheter.

## Methods

### Chemicals

All chemicals were purchased from Sigma Aldrich (Saint Louis, MO, USA) unless otherwise stated. For the phantom experiment, two sets of samples were prepared in either aqueous solutions or 2% agarose gel. The D_2_O (99.9%) was mixed with PBS (pH = 7.4) at the final concentrations (v/v) of 5%, 10%, 20%, 40%, 60% and 80%. All samples were prepared freshly and transferred to 5 mm NMR tubes prior to MRI measurement.

### Animals and endovascular cannulation

All procedures were approved by our institutional Animal Care and Use Committee and in accordance with guidelines for the care and use of laboratory animals. Sprague-Dawley rats (male, 200-250 g, Charles River, n = 6) were used to demonstrate the ability D_2_O-MRI to monitor the cerebral IA injection by a means of carotid artery catheterization. The surgical procedures were performed as previously described 11. Briefly, the animals were anesthetized under 2% isoflurane and positioned supine. The common carotid artery (CCA) bifurcation was dissected, and the occipital artery was coagulated. The external carotid artery (ECA) and pterygopalatine artery (PPA) were temporarily ligated with 4-0 silk sutures. A small arteriotomy in the CCA was made and a catheter (VAH-PU-C20, Instech Solomon Inc.) connected to #30 PTFE tubing was introduced into the internal carotid artery (ICA) via the arteriotomy. The intra-arterial catheter was secured with the animal during MRI scans.

The same catheterization procedures were performed for cerebral IA injection in brain tumor-bearing mice. In brief, six C57BL6 mice (female, 5-6 weeks) were stereotactically injected with 5 x 10^4^ murine glioma CT2A cells. The injection coordinates were -2.0 mm for the anterior-posterior (from Bregma), 1.5 mm for medial-lateral (from midline), and -3.0 mm for dorsal-ventral (from the surface of skull) axes, respectively. Orthotopic brain tumors were allowed to grow ~ 7 days to reach the desirable size (~2-3 mm in diameter). Prior to MRI, three mice were randomly selected to, under anesthesia, put a microcatheter (PE-8-100, SAI Infusion Technologies) in proximal ICA via a small arteriotomy from CCA. After the intra-arterial catheter was secured using two purse-string suture ties applied around CCA, mice were transferred to the MRI scanner. The rest of the mice (n = 3) were injected with 0.6 mL IgG-FITC (1.5 mg/mL) via tail vein and euthanized two hours later to collect brains for *ex vivo* fluorescence imaging.

For cerebral IA injection into the anterior cerebral artery in a canine (male greyhound dog, weight ~ 25 kg, n = 1), a 5-French femoral arterial sheath was surgically introduced. A 5-French Glide catheter was then advanced over a 0.035-inch guide wire and the right anterior cerebral artery was selectively catheterized using a 1.7-French microcatheter was then advanced over a 0.014-inch microwire under X-ray angiography roadmap guidance.

### MRI

*In vitro* MRI was performed on an 11.7 T Bruker Avance system (Bruker, Ettlingen, Germany) equipped with a 15 mm sawtooth RF coil. All measurements were conducted at room temperature (20 ^o^C). T_1_ and T_2_ maps were acquired according to our previously published procedures 12. In brief, longitudinal (T_1_) relaxation times of the samples were assessed using a Rapid Acquisition with Relaxation Enhancement (RARE) -based saturation recovery sequence with eight TR times ranging between 200 ms to 15,000 ms (TE = 4.3 ms and RARE factor = 4, central encoding). T_2_ maps were acquired using a modified RARE pulse sequence (TR/TE = 25000/4.3 ms and RARE factor = 16) with a Carr-Purcell-Meiboom-Gill (CPMG) T_2_ preparation module. The T_2_ preparation period consisted of an element of CPMG pulse train with t_CPMG_ = 10 ms, and a total of 16 CPMG loops (2 to 1024) were used, corresponding to echo times = 20 ms to 10.24 sec. Other imaging parameters were: 256x256 acquisition matrix with a spatial resolution of *c.a.* 50x50 µm^2^, and slice thickness of 1 mm.

**Figure 1:**
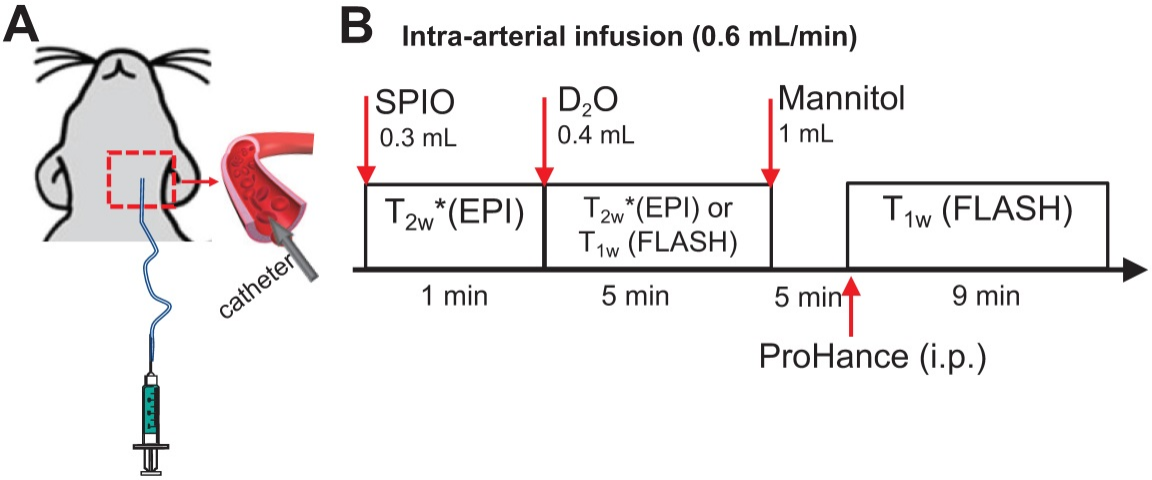
**Schematic of MRI studies of intracarotid infusion of mannitol in the rat brain**. **A**) A catheter was inserted into the ICA for IA injection of SPIO, D_2_O and mannitol. **B**) Timeline of MRI study. The rat was sequentially scanned after IA injection of SPIO and then D_2_O. After the mannitol injection, the animal was removed from the scanner to receive intra-peritoneal (*i.p.*) injection of ProHance (Gadoteridol, 0.1 mmol/kg) and T_1w_ images were acquired.

Rat and mouse MRI was performed on a horizontal bore 11.7 T Bruker Biospec system using a 72 mm quadrature volume resonator as a transmitter, and a four-element (2×2) phased array coil as a receiver. The timeline of *in vivo* MRI study is illustrated in **Figure 1**. Briefly, the perfusion territory was assessed by both SPIO- and D_2_O- MRI. To precisely determine the perfusion territory, SPIO and D_2_O were infused at the same flow rate as mannitol. Then, BBB opening (BBBO) was induced by infusion of 1 mL mannitol (25%, Hospira) *via* the IA route at the speed of 0.6 mL/min. Five minutes after mannitol injection, BBBO was assessed by Gd-based DCE MRI.

For SPIO-MRI, 300 µL ferumoxytol (0.3 mg/mL) was injected through an IA catheter, and dynamic T_2w_* images were acquired using a multi-slice echo planar imaging (EPI) sequence with TR/TE = 2000/9.7ms, segment factor = 2, bandwidth = 455kHz, slice number = 15, slice thickness = 1mm, a matrix size of 128×128 within a FOV of 3×3 cm^2^ 13.

For Gd-MRI, a bolus of Prohance (0.1 mmol/kg) was injected intraperitoneally, and dynamic T_1w_ images were acquired using a FLASH sequence with TR/TE = 100 ms/3 ms, flip angle = 25°, average number = 1, slice number = 11, slice thickness = 1 mm, a matrix size of 128×128 within a FOV of 3×3 cm^2^. The acquisition time for each scan is 19 sec.

D_2_O was IA injected at the rate of 0.6 mL/min and the total injection volume was 0.4 mL and D_2_O-MRI was acquired using the same EPI (n = 3) or FLASH sequences (n = 3), which would permit the direct comparison of D_2_O MRI measures with those of SPIO- or Gd-CE MRI, respectively.

The same MRI methods were used for mouse imaging (n = 3), except a series of bolus injections (duration = 20 s) of D_2_O at different infusion rates of 50, 100, 150, or 200 µL/min (total injection volume = 16.7- 66.7 µL) was investigated for the optimal perfusion to the tumor region. At each infusion rate, seven D_2_O MR images were acquired starting at 40 s before injection till 74 s after the stop of injection (total acquisition time= 2 min 14 s). Using the optimal rate determined by D_2_O MRI, a mixture (0.6 mL) of 25% mannitol and IgG-FITC (1.5 mg/mL) was then administered. Two hours later, mice were sacrificed, and brains were harvested and fixed in 4% paraformaldehyde solution for *ex vivo* fluorescence imaging.

Dog MR imaging was performed on a clinical 3T Siemens Trio using a quadrature head coil. The dynamic T_2_*_w_ images during IA D_2_O and SPIO (2mL/min) were acquired using a single shot GE-EPI (TE = 36 ms, TR = 3000 ms, FOV = 180x180 mm^2^, matrix size = 192x192, slice thickness= 2.5 mm, and acquisition time = 3 s and 50-100 repetitions). Two mL of 25% mannitol over 1 min was administrated thereafter at the same rate as D_2_O injection. Five minutes after IA mannitol, a bolus of Magnevist (gadolinium, 0.125 mmol/kg) was injected intravenously. Standard Gd-CE (Gd-contrast enhancement) images were acquired then.

### Fluorescence imaging

The uptake of IgG-FITC in the fixed mouse brains was measured using a Spectrum/CT IVIS® *in vivo* imaging system (Ex/Em = 500/540 nm) and analyzed using the Living Image® processing software from the manufacturer (PerkinElmer, Waltham, MA).

### Data processing

All experiment data were processed by either custom-written scripts in MATLAB (R2020a, Mathworks, Natick, MA, USA) or ImageJ (version 1.51, NIH, https://imagej.nih.gov/ij/, Bethesda, MD, USA). For* in vitro* data, using ROI masks that were manually drawn on a T_2w_ image, mean ROI values were calculated and plotted. The proton density (PD) of each sample was estimated using the mean ROI value of the final image of the saturation recovery MRI, where TR = 15 sec and effective TE = 4.3 ms.

T_1_ relaxation times were estimated by fitting the ROI values to Eq. 1,


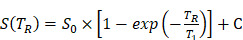
Eq. 1

where *S*(*T*_R_) is the MRI signal at each *T*_R_ time, and the theoretical maximal MRI signal *S*_0_, T_1_ times, and constant C are the parameters to be estimated.

The R_1_ relaxivity (r_1_) of each compound was subsequently estimated by fitting water proton relaxation rates (R_1_ = 1/T_1_) as a linear function of D_2_O concentration (Eq. 2).


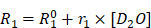
Eq. 2

where *R*_1_^0^ is the water intrinsic *R*_1_ relaxation rate, a fitting parameter in our study.

T_2_ relaxation times were estimated by fitting ROI values to Eq. 3,


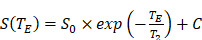
Eq. 3

where *S*(*T*_E_) are the MRI signal at each CPMG *T*_E_ time, and the theoretical maximal MRI signal *S*_0_, T_2_ time, and constant C are the parameters to be estimated.

The R_2_ relaxivity (r_2_) of each compound was subsequently estimated by fitting R_2_ relaxation rates (R_2_ = 1/T_2_) as a linear function of D_2_O concentration (Eq. 2).


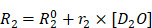
Eq. 4

where *R*_2_^0^ is the water intrinsic *R*_2_ relaxation rate, a fitting parameter in our study.

For *in vivo* data, we calculated the pixel-wise maximum signal change map (∆SI^max^) after injection of SPIO or D_2_O using the Matlab function max ([SI^post^(t)- SI^pre^]/SI^pre^). The Gd-contrast-enhanced (CE) map was calculated by comparing the post-contrast image at 9 min with that of before injection.

The area affected by the first pass of transcatheter injection, namely perfusion territory, was delineated using a histogram-based analysis as described previously for SPIO 14 and Gd agents^13^. In brief, for each animal, a histogram was plotted to represent the frequency distribution (percentage of voxels, y-axis) of the level of signal change (x-axis) within the entire brain, which was then fitted into a 2-Gaussian distribution using custom-written Matlab scripts. A cut-off of signal change was chosen at where the overlap between two Gaussian functions was minimum. Pixels with signal changes larger than the cut-off were assigned as the perfusion territory.

### Statistical analysis

All statistical analyses were conducted using Matlab and GraphPad Prism (version 7, GraphPad Inc, San Diego, USA). The contrast maps generated by D_2_O, SPIO, and Gd-DOTA were compared using the non-parametric Spearman's rank-order correlation analysis. Non-parametric tests were used because the normality of data distribution was not passed the D'agostino-Pearson test. The Student's t-test was used to compare the D_2_O contrast changes in the tumor at different infusion rates and the difference between the mean fluorescence intensities in the mouse brains administered using MRI-guided IA injection and those by IA injection. In all these analyses, p-value < 0.05 was regarded as statistically significant.

## Results

### D_2_O as a negative MRI contrast medium

**Figure 2:**
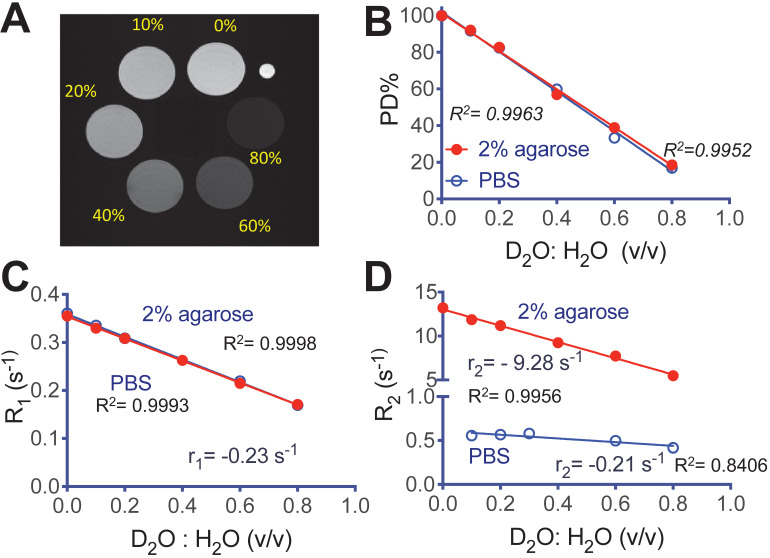
** MRI of deuterated aqueous PBS solutions and agarose (2%)**. **A**) Representative proton densities (PD)-weighted image (TR/TE = 15,000/4.3 ms) of D_2_O-H_2_O samples in 2% agarose gel, where a small capillary (OD = 1 mm) containing PBS was used as a marker for sample position. **B**) Proton densities, **C**) R_1_ relaxation rates, and **D**) R_2_ relaxation rates of aqueous (blue) and gel (red) samples prepared at different D_2_O: H_2_O ratios.

We first characterized the MRI contrast of D_2_O in aqueous solution and 2% agarose gel. **Figure 2A** shows a representative MR image of D_2_O agarose gel samples at concentrations from 0 to 0.8 (D_2_O : H_2_O volume fraction) that were acquired using an 11.7T preclinical scanner. The negative effect of D_2_O on proton density (PD) contrast is linearly proportional to its concentration (R^2^ = 0.9952 and 0.9963, in solution and gel respectively, **Figure 2B**), attributed to the proton replacement effect. D_2_O also has relatively small but observable effects on water T_1_ and T_2_ contrast. At the field strength used in our study (*i.e.*, 11.7T), the r_1_ relaxivities per D_2_O fraction (v/v) were estimated to be -0.230 s^-1^ (R^2^ = 0.9998) and -0.236 s^-1^ (R^2^ = 0.9993) in solution and gel, respectively (**Figure 2C**). The r_2_ relaxivities per D_2_O fraction (v/v) were estimated to be -0.211 s^-1^ (R^2^ = 0.8406) and -9.28 s^-1^ (R^2^ = 0.9956) in solution and gel, respectively (**Figure 2D**). The negative sign of relaxivity denotes the tendency of D_2_O to decrease the R_1_ and R_2_ relaxation rates (increase T_1_ and T_2_ relaxation times) of water, as opposed to commonly used MRI relaxation contrast agents. It should be noted that, as indicated by the simulations (**Figure S1**, Supporting Information), the T_1_/T_2_ effects are not negligible and, depending TR/TE parameters, may cause errors if simply interpreting the signal decrease (∆SI) as the concentration of D_2_O.

**Figure 3:**
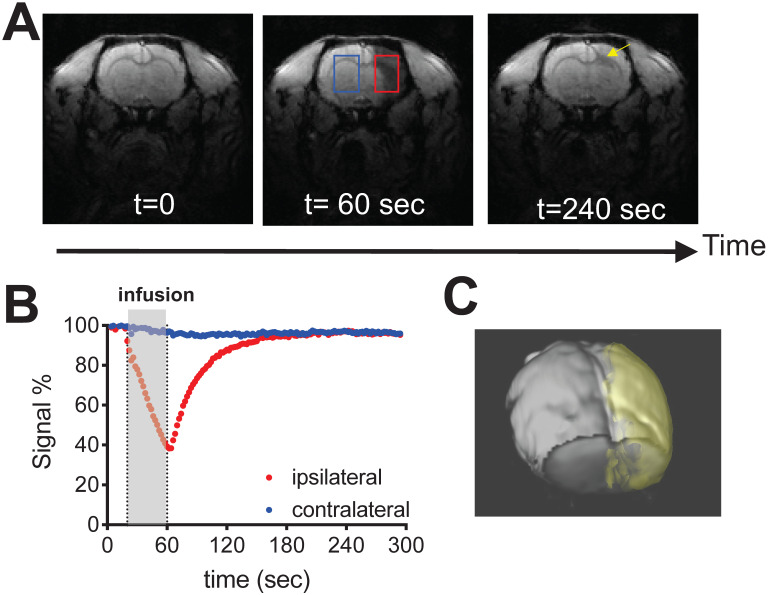
** Dynamic D_2_O-enhanced MRI in a representative rat brain after intraarterial injection of 0.6 mL D_2_O. A**) MR images before (t = 0), during (t = 60) and post-injection (t = 240 sec). **B**) Dynamic signal changes in two representative ROIs that were manually drawn in the ipsilateral (red) and contralateral (blue) hemispheres. **C**) 3D visualization of the perfusion territory (gold).

As shown in **Fig. 3** and the **supp. video S1**, IA infusing of D_2_O (injection rate = 0.6 mL/min, total volume = 0.4 mL) to the rat brain resulted in a rapid signal decrease in the ipsilateral brain hemisphere (red ROI), attributed to the proton replacement effect of locally high concentration of D_2_O. As soon as the infusion was stopped, MRI signal recovered exponentially, reflecting quick washout of D_2_O from the tissue 15. At the end of the MRI scan, ~ 4 minutes after the stop of infusion, the MRI signal recovered back to 95.3% of its baseline level. In comparison, a much slower signal reduction was observed in the contralateral hemisphere (**Figures 3A, B**, blue ROI), *i.e.,* ∆SI ~ 4.0% by the end of MRI scan, attributed to circulating D_2_O in the bloodstream. The signal changes of the two ROIs reached equilibrium approximately at 2 minutes after the infusion was stopped. It should be noted that in certain regions (**Figure 3A**, yellow arrow), D_2_O clearance was quite slow, *i.e.*, remaining ∆SI = 14.5% at the end of the study, indicating that a significant amount of D_2_O entered and remained in the interstitial and intracellular spaces in these regions. Using the ∆SI% at the time of infusion stop, we can determine the perfusion territory that the infused solution can reach (**Figure 3C**). It should be noted that due to the T_1_/T_2_ relaxation effects as shown in **Figure S1**, ∆SI is not necessarily the same as D_2_O concentration. Indeed, assuming that majority of D_2_O still remained in the blood (where T_10_/T_20_ = 2800/46 ms 16), with the acquisition parameters used to acquire **Figure 3** (TR/TE = 100/3 ms), the concentrations of D_2_O to generate 4.0% (contralateral) and 4.7% (ipsilateral) signal decrease are estimated to be 2.5% and 2.9%, respectively.

### Delineation of perfusion territory using D_2_O

**Figure 4:**
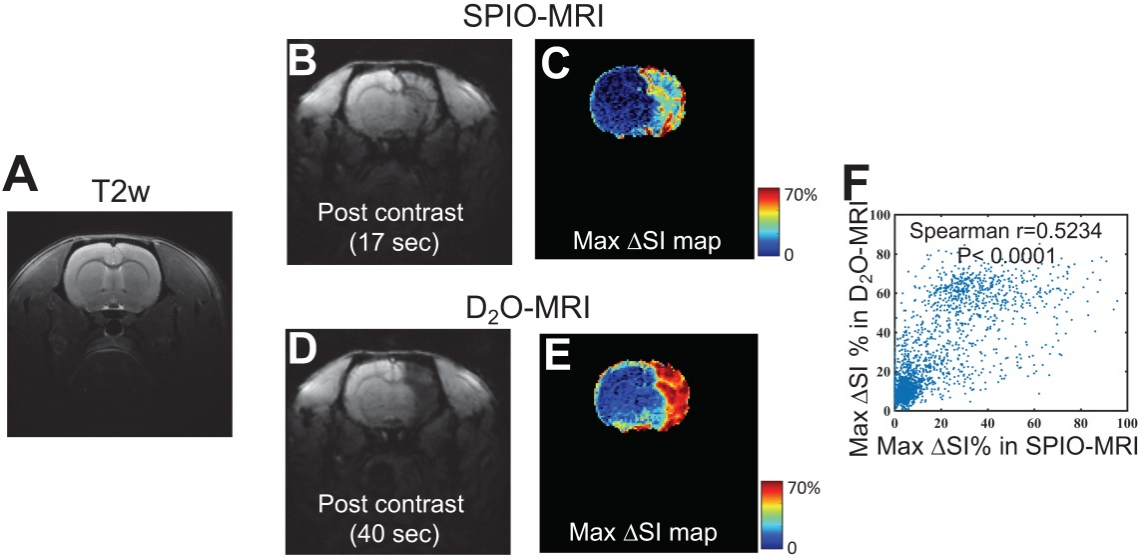
** Comparison of the perfusion regions measured by SPIO-MRI and D_2_O-MRI. A**) T_2w_ reference image.** B**) T_2_*_w_ image and **C**) Maximum contrast-enhancement map by SPIO infusion. **D**) T_2_*_w_ image and **E**) Maximum contrast-enhancement map by D_2_O infusion. **F)** Correlation between SPIO- and D_2_O- enhanced MRI. The Spearman correlation coefficient (r) was determined to be 0.5234.

**Figure 4** shows the comparison between the perfusion *territories* determined by SPIO- and D_2_O-MRI in a representative rat (**Figures 4B,D**), revealing similar contrast enhanced regions. This is further confirmed by comparing the maximal contrast-enhancement maps (**Figure 4C**) acquired by the two methods, with a good pixel-wise correlation (Spearman coefficient = 0.5234, P < 0.0001, **Figure 4F**) and good overlap between the contrast-enhanced regions. As shown in **Figure S2**, all animals showed good agreement between the territories determined by the two methods (r = 0.5692 and 0.5585, for the other two rats), which implies that, even there are fundamental differences between D_2_O and SPIO nanoparticles, D_2_O can also be used as an alternative to SPIO for delineating the perfusion territory of endovascular injection.

### Prediction of mannitol-induced BBB opening using D_2_O-MRI

**Figure 5:**
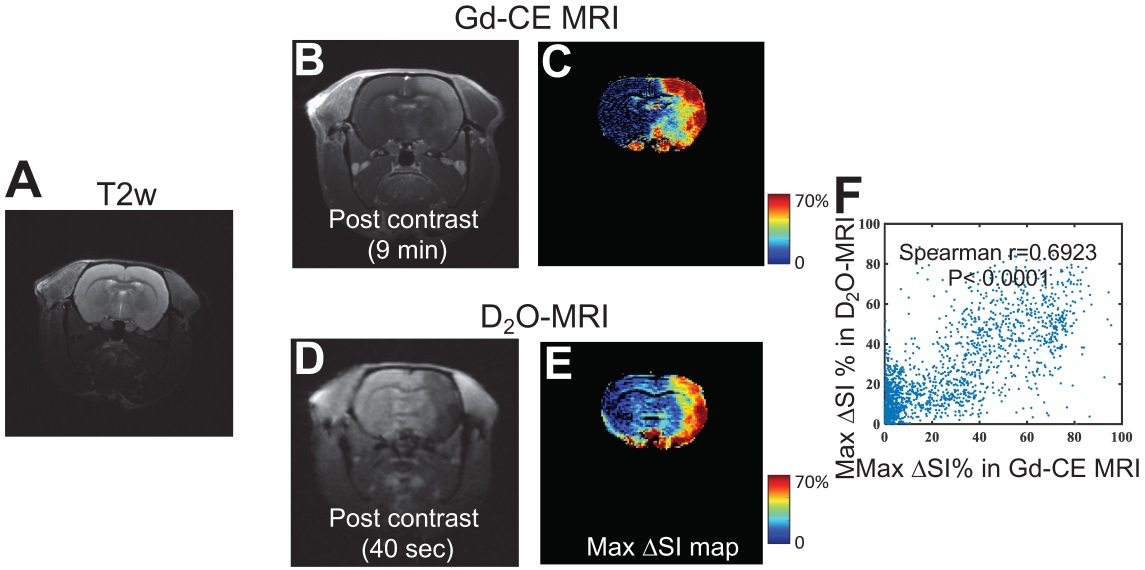
** Comparison of the BBB opening regions predicted by D_2_O-MRI and the BBB-disrupted region measured by Gd-CE MRI. A**) T_2w_ reference image.** B**) T_1w_ image and **c**) Maximum contrast-enhancement map by Gd-CE MRI. **D**) T_1w_ image and **E**) Maximum contrast-enhancement map by D_2_O. **F)** Correlation between the two measures. The Spearman correlation coefficient (r) was determined to be 0.6923.

We then investigated the ability of D_2_O-MRI to predict the effective regions in response to a transcatheter infusion procedure, in particular, in the present study, the regions where BBB will be opened by mannitol. As depicted in **Figure 1**, D_2_O-MRI was performed before mannitol infusion, and Gd-enhanced MRI was carried out after mannitol treatment to assess the resulted BBBO. The comparison of Gd-MRI and D_2_O-MRI is shown in **Figures 5B-E**, revealing the contrast-enhanced areas by the two methods were in good agreement, *i.e.*, Spearman coefficient = 0.6923 (P < 0.0001, **Figure 5F**). As shown in **Figure S2**, a good correlation was found between the territories determined by the two methods in all animals (r = 0.6558 and 0.5817, for the other two rats). Given Gd-CE MRI is a commonly used method to assess BBBO after intervention, our results imply that, even there are fundamental differences between D_2_O and Gd agents, D_2_O-MRI may also be used as the alternative to Gd-CE MRI by predicting the BBBO reliably before the actual injection of mannitol.

### D_2_O-MRI guided drug delivery to brain tumors in a mouse model

**Figure 6:**
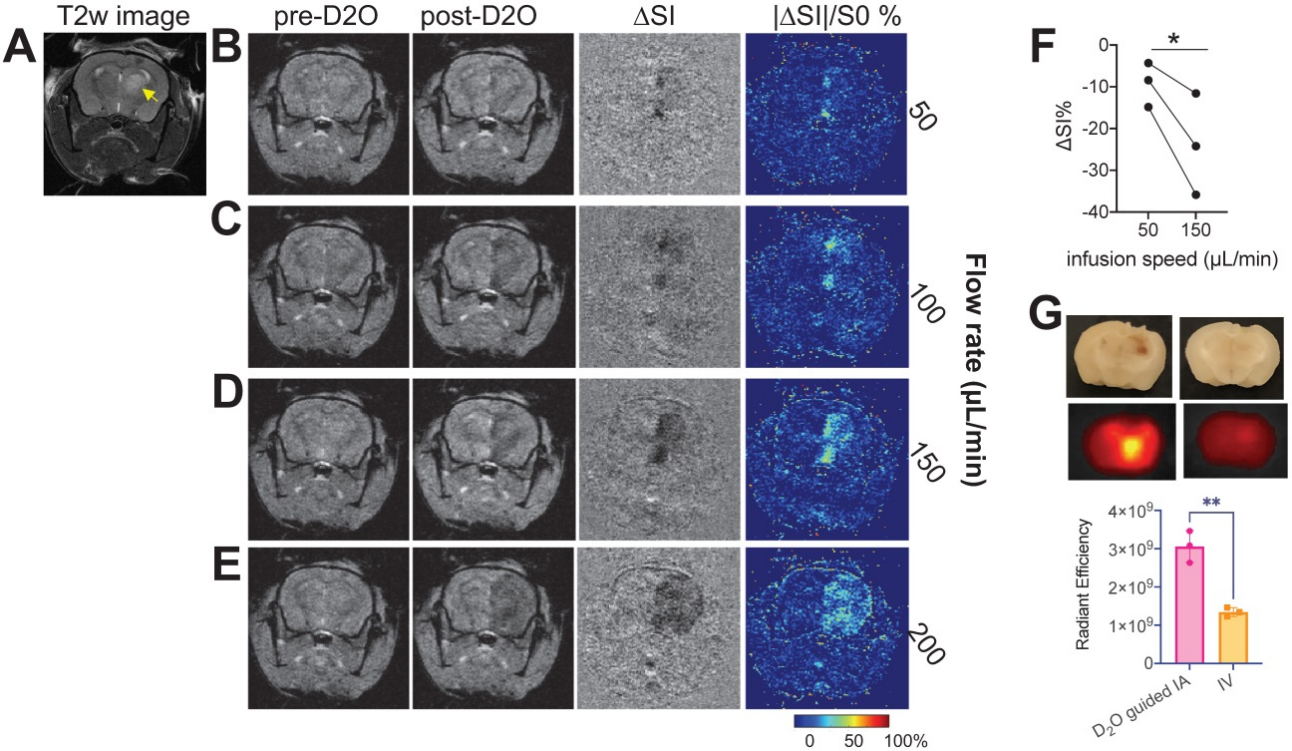
** D_2_O-MRI guided neurointervention drug delivery to a brain tumor in a representative mouse**. **A**) T_2w_ image showing the location of the tumor (yellow arrow). Pre-D_2_O images, post-D_2_O images, contrast maps (∆SI = S^post^- S^pre^), and maps of relative signal change (%) with D_2_O injected at 50 µL/min (**B**), 100 µL/min (**C**), 150 µL/min (**D**), and 200 µL/min (**E**), respectively. **F**) Bar plots showing the comparison between the mean MRI signal changes in tumor regions of three mice using two different flow rates (P = 0.0382, paired Student's t-test). **G**) *ex vivo* fluorescence imaging of IgG-FITC accumulated in the mouse brains with the IgG injected either by D_2_O-guided IA injection or by IV injection (P = 0.0023, unpaired Student's t-test).

We then demonstrated the ability of D_2_O MRI to guide interventional drug delivery in an orthotopic mouse brain tumor model. For transcatheter injection, besides the location of the catheter, injection rate is another important parameter determining the area that the injected agent can reach. Our results showed that, when injected at slow rates (*i.e.,* 50 and 100 µL/min), only a small portion of the tumor could be perfused as revealed by the D_2_O MRI (**Figures 6B,C**), indicating a low delivery efficiency. Increasing infusion rate significantly increased the delivery efficiency to the tumor (**Figures 6D,E**). However, when the rate was too high (200 µL/min, **Figure 6E**), overwhelming perfusion in the brain parenchyma was observed, which may cause unwanted adverse effects. Using D_2_O-MRI as imaging guidance, we determined that 150 µL/min to be the optimal rate for drug delivery to the tumors without serious off-target delivery. D_2_O MRI revealed that all animals (n = 3) showed a significantly augmented perfusion in the tumor region as compared to that by a low rate (P = 0.0382, paired Student's t-test, **Figure 6F**) when this optimal rate was used. The optimized drug delivery was confirmed by fluorescence imaging using fluorescent IgG-FITC (resembling antibody drugs, which cannot permeate the intact BBB). The quantitative analysis of *ex vivo* fluorescence imaging of the excised mouse brains showed a two-fold higher drug delivery efficiency (as quantified as total radiant efficiency (photons/s)/(μW/cm^2^)) was achieved to deliver antibody IgG to the tumor-bearing brain compared to the traditional intravenous (IV) injection route (P = 0.0023, unpaired Student's t-test).

### First-in-dog 3T D_2_O-MRI study

**Figure 7:**
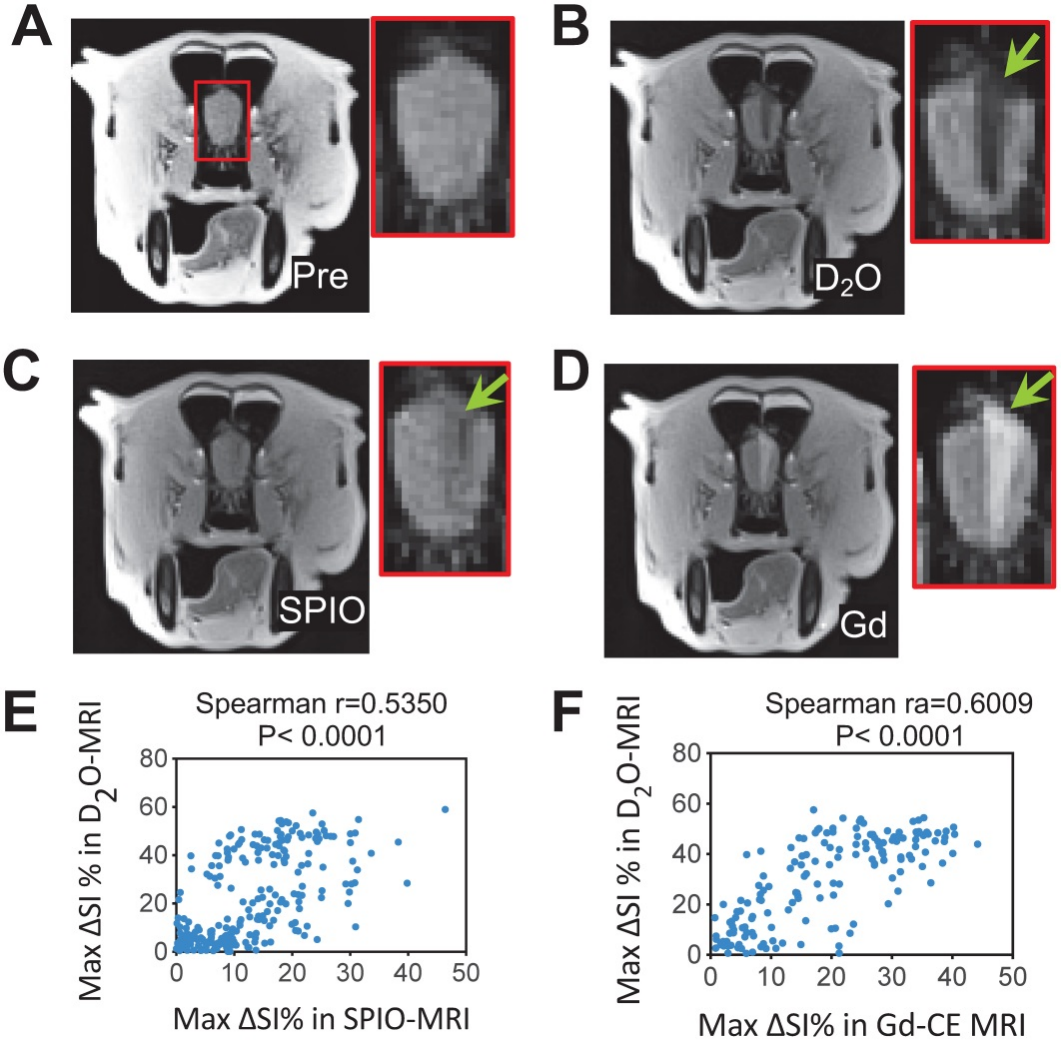
** 3T D_2_O MRI guidance for interventionally hyperosmotic BBBO in a dog**. **A**) T_2w_ image pre-injection. **B**) D_2_O MRI contrast-enhancement map. **C**) SPIO MRI contrast-enhancement map. **D**) Gd-contrast-enhancement map after the administration of mannitol. **E)** Correlation between SPIO- and D_2_O- MRI. The Spearman coefficient (r) = 05350. **F)** Correlation between Gd- and D_2_O- MRI. The Spearman coefficient (r) = 0.6009.

Finally, we performed a 3T D_2_O-MRI in a canine model for predicting interventional BBBO areas (**Figures 7 and S3**). As can be seen in **Figures 7B-C**, both SPIO and D_2_O MRI methods were able to assess the perfusion territories, but the regions revealed by D_2_O MRI appeared larger than SPIO MRI. A moderate correlation (Spearman r = 0.5350, P < 0.0001) was found between the hypointense regions caused by D_2_O and SPIO (**Figure 6E**). Following the infusion of mannitol, areas of BBBO were visualized by Gd-CE MRI (**Figure 6D**). The hypointense area of D_2_O correlated well with the hyperintense area shown in Gd-CE MRI (Spearman r = 0.6009, P < 0.0001,** Figure 6F**), indicating D_2_O MRI can be used to guide interventional BBBO conducted on 3T MRI scanners. The predictive power of D_2_O MRI was even better than SPIO MRI, which only showed a relatively poor correlation with Gd-CE MRI (Spearman r = 0.4365, P < 0.0001). This pilot 3T study demonstrates the feasibility of D_2_O-MRI on clinical MRI scanners and future studies are warranted to fully test and validate the clinical utility of D_2_O MRI.

## Discussion

Our study demonstrated that the negative contrast generated by D_2_O can be used as a useful contrast medium to assess the spatial and temporal distribution of the injected solution in the brain, providing a new means to guide endovascular neurointervention. D_2_O-MRI has the capacity for accurately determining the perfusion territory and predicting the affected areas and treatment responses, allowing for real-time guiding endovascular interventions such as IA drug delivery targeted to a specific region in the brain.

Deuterium is a non-radioactive isotope of hydrogen with a natural abundance of 0.0156%. As a stable and enriched isotopic form of ^1^H water, D_2_O has been used in clinical tests and treatments with few adverse effects 17. For example, deuterium-dilution technique is the gold standard to measure total body water space 18 and body composition 19, which is even safe for children. Numerous studies showed the low toxicity of D_2_O even at the level of 20% of total body fluids over a short period 2, 20. Unlike previous studies required the *i.v.* injection of D_2_O at a relatively high dose (*e.g.,* 20 mL/kg 21), our study employed a relatively low dose (*i.e.*, 1.6 mL/kg). Indeed, this dose level only accounts for ~3.2% plasma concentration (total injection volume of D_2_O = 0.4 mL, assuming the total blood volume of rats is ~12.5 mL). Considering the high contrast level achieved in the present study, the injection volume can be further decreased substantially. Hence, D_2_O-MRI can be considered as a highly safe approach for accomplishing image-guided endovascular neurointervention.

D_2_O can be directly detected by ^2^H NMR or MRI, an approach explored for measuring blood flow and perfusion in normal organs 21-24 and tumors 15, 23, 25, 26. However, its wide application is hindered by low sensitivity (*i.e.*, ~6.5 times lower than ^1^H) and the need for special hardware. In ^1^H NMR or MRI, D_2_O is invisible and will reduce the detectable MR signal when mixing with water in a concentration-dependent manner. While D_2_O produces ^1^H MRI contrast mainly through changing the proton density of water by the so-called proton replacement effect, it also has observable effects on the T_1_ and T_2_ relaxation times of water. Of note, the r_2_ of D_2_O in agarose gel (*I.e.*, -9.28 s^-1^) was found much larger than that in aqueous solution (*i.e.*, -0.21 s^-1^), whereas the r_1_ of D_2_O was largely unaffected (-0.230 and -0.236 s^-1^ in solution and gel, respectively). The much stronger T_2_ effect of D_2_O in the agarose gel can be explained by the fact that its effect to weaken the dipole-dipole interaction between protons would become more pronounced when the mobility of water molecules becomes slower due to either a higher amount of bound water on the surface of macromolecules 27 or a slow diffusion rate 28. Our observation is consistent with previous studies 28, 29. For example, Zhong et al. 29 reported the r_2_ relaxivities of D_2_O (at 7T) of -0.2 s^-1^ and -5.3 s^-1^ in pure solution and rat liver, respectively.

The negative MRI contrast of D_2_O was used to measure water permeability across cell membranes *in vitro* 30 and cerebral blood flow *in vivo* 10. For the later application, D_2_O was injected through the tail-vein at a dose of 2 mL/100g body weight, and approximately 5% MRI signal decrease was generated in the brain 10. In comparison, we explored the application of D_2_O as a contrast medium for guiding IA drug delivery, in which D_2_O was injected directly into the blood vessels of interest. As a result, the contrast was remarkably high (> 60% in the areas close to the catheter), which is sufficient for rendering the perfusion territory reliably. Indeed, in the present study, we have successfully demonstrated the utility of D_2_O MRI in improving the delivery of antibodies to the targeted brain tumor using an image-guided neurointervention approach. Based on our findings, we foresee the great potential of D_2_O-MRI in endovascular neurointervention.

During an endovascular intervention, the affected areas are largely determined by the transcatheter perfusion territory, which is determined by the location of catheter and infusion rate. The assessment of perfusion territory is crucial for the precision and effectiveness of an intravascular intervention. SPIO has been previously used to detect the perfusion territory in the brain and to guide IA injection 31. Our present study revealed a good correlation between the perfusion territory determined by SPIO-MRI and that by D_2_O-MRI. However, it should be noted that perfusion territories by the two methods are only moderately correlated. The discrepancy is not a surprise as D_2_O is a small molecule and to which BBB is permeable (via an active water transport mechanism 32), whereas SPIO is a nanoparticle and to which intact BBB is completely impermeable. Moreover, SPIO particles have a well-known spatial “blooming effect” in the T_2_*w images, which may lead to overestimation of perfusion area. This effect is caused by the quick diphase of diffusing water molecules by the SPIO-generated local B_0_ disturbances/inhomogeneities 33. As D_2_O behaves just like a water molecule and causes no B_0_ distortion, it would not cause a blooming effect. The perfusion region measured by D_2_O is hence more accurate. Indeed, our 3T study on a canine model revealed that D_2_O-MRI provided a better prediction of the BBBO region than SPIO-MRI, suggesting D_2_O is an excellent contrast medium for rendering the perfusion territory and guiding IA drug delivery.

One potential limitation of using D_2_O MRI to guide endovascular intervention is that D_2_O is a diffusible tracer that won't restrictively stay in the bloodstream after injection. Hence, the accurate determination of its concentration in the intravascular (or extravascular) compartment will require compartmental pharmacokinetic analysis 15, 34. Another potential limitation is the slow washout kinetics of D_2_O in some regions. This can prevent D_2_O from being used repeatedly in a short time interval. Reducing the injection volume may partially overcome this challenge, and further studies to optimize the injection volume are warranted. Similar to SPIO, D_2_O generates a negative contrast on MR images. Generally speaking, positive contrast (*e.g.*, Gd agents) is more preferable because the extent of contrast enhancement can be higher than 100% by a positive contrast agent, whereas maximum change by a negative contrast agent (*e.g.*, SPIO and D_2_O) is 100%. Moreover, the darkened regions by a negative contrast agent may not be distinguishable from voids in the image caused by other effects, such as image artifacts or intrinsic hypointense tissues. Fortunately, as demonstrated in the present study, when a dynamic imaging scheme is used, the post-contrast images are compared with the baseline image, allowing easy discrimination between the darkened area generated by D_2_O easily and intrinsic hypointense regions. Finally, it should be noted that the quantification of D_2_O could be confounded by its effects on water T_1_ and T_2_ relaxation times. Depending on the TR and TE times and intrinsic tissue T_1_ and T_2_ times, the MRI signal change (contrast) caused by D_2_O may deviate significantly from the pure proton replacement effect. The T_2_ effect is even more pronounced when D_2_O enters tissues than in the blood. Therefore, the accurate quantification of D_2_O requires taking the relaxation effects into account. If possible, long TR and short TE should be used to reduce the errors.

## Conclusion

In summary, our study showed that D_2_O is a negative MRI contrast medium suitable for guiding endovascular neurointervention. Using a high field 11.7T and low field 3T ^1^H MRI scanners, we demonstrated the ability of D_2_O MRI to accurately visualize perfusion territory and predict BBBO regions in different animal models. The D_2_O-MRI is a safe approach with a good quantitative ability, and hence may be useful for a variety of endovascular procedures.

## Supplementary Material

Supplementary figures.

Supplementary video/movie.

## Abbreviations

BBB: blood brain barrier
D2O: deuterium oxide
IA: intra-arterial
BBBO: BBB opening
ROI: region of interest
SPIO: superparamagnetic iron oxide particles
