# Supplementary material for: Deuterium oxide as a contrast medium for real-time MRI-guided endovascular neurointervention
Source: Theranostics. 2021 Apr 15;11(13):6240–50. doi: 10.7150/thno.55953 (PMC8120199; doi:10.7150/thno.55953)
Supplement: Supplementary file 1 — Supplementary figures. [file thnov11p6240s1.pdf]

Supporting information

Deuterium oxide as a contrast medium for real-time MRI-guided endovascular neurointervention

Lin Chen[1,2#], Jing Liu[3,2#], Chengyan Chu[4#], Zheng Han[2,5#], Nirhbay Yadav[2,5], Jiadi Xu[2,5], Renyuan Bai[6], Verena Staedtke[7], Monica Pearl[2], Piotr Walczak[4], Peter van Zijl[2,5], Miroslaw Janowski[4], Guanshu Liu[2,5 *]

1. Department of Electronic Science, Fujian Provincial Key Laboratory of Plasma and Magnetic Resonance, School of Electronic Science and Engineering, National Model Microelectronics College, Xiamen University, Xiamen, Fujian, China

2. Russell H. Morgan Department of Radiology and Radiological Science, Johns Hopkins University School of Medicine, Baltimore, MD, USA

3 The First Affiliated Hospital of Jinan University, Guangzhou, Guangdong, China

4. Department of Diagnostic Radiology and Nuclear Medicine, University of Maryland, Baltimore, MD, USA

5. F.M. Kirby Research Center for Functional Brain Imaging, Kennedy Krieger Institute, Baltimore, MD, USA

6. Department of Neurosurgery, Johns Hopkins University, Baltimore, MD, USA

7. Department of Neurology, Johns Hopkins University, Baltimore, MD, USA

[#] These authors contributed equally

* Corresponding author:

Guanshu Liu, Ph.D.

Email: guanshu@mri.jhu.edu

Supplementary video 1: Dynamic D_2O-enhanced MRI

S1. Simulations to estimate the potential errors caused by the R_1/R_2 effects of D_2O at different concentrations.

The normalized MRI signals in the brain and blood with different [D_2O] were computed by

$$S\,(D_2O) = PD * (1 - exp(-TR * R_1)) * exp(-TE * R_2)$$

Where PD is the normalized proton density and equals 1-*[D_2O], R_1= R_{10}+ r_1 [D_2O], and R_2= R_{20}+ r_2 [D_2O].*

In the case where the T_1/T_2 effects of D_2O is ignored, the ratio of the MRI signal before and after D_2O injection is used to measure the concentration of D_2O ([D_2O]$_{measure}$):

$$[D_2O]_{measure} = 1 - \frac{PD(D_2O)}{PD_0} = 1 - \frac{S}{S0} = 1 - \frac{[D_2O]_{true}(1 - exp(-TR * R_1)) * exp(-TE * R_2)}{(1 - exp(-TR * R_{10})) * exp(-TE * R_{20})}$$

Scenario 1: D_2O is located is refined in the blood, and images are acquired using 1) FLASH sequence (TR/TE = 100/3 ms) at 11.7T, 2) EPI sequence (TR/TE = 1000/10 ms) at 11.7T, and 3) EPI (TR/TE =3000/36 ms) at 3T.
The values of blood T1 and T2 were 2800/46 ms at 11.7T as reported by Lin [1] and 1932/275 ms at 3T as reported by reported by Stanisz [2]. While we did not measure the r_2 of D_2O in the blood experimentally, we adapted the reported value of ~ -0.3 sec^{-1} by Zhong for BSA solution.

Scenario 2: D_2O is located in the brain parenchyma. The same sequence parameters as in scenario1 are used. The values of blood T1 and T2 were 1900/33.8 ms at 11.7T as reported by de Graff [3], and 1084/69 ms at 3T as reported by reported by Stanisz [2].

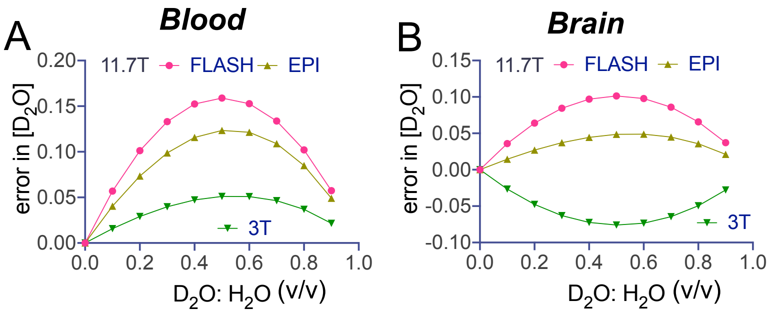

Figure S1. Simulations showing the measurement errors in D_2O concentration as a function of nominal D_2O concentration in the A) Blood and B) Brain using the acquisition parameters used in the present study.

The simulation showed that the errors can be substantial. For example, the measured error can be as high as 0.15/0.5= 30% when D2O is in the blood and a FLASH sequence (TR/TE = 100/3 ms) is used. If possible, long TR and short TE should be used to reduce the errors.

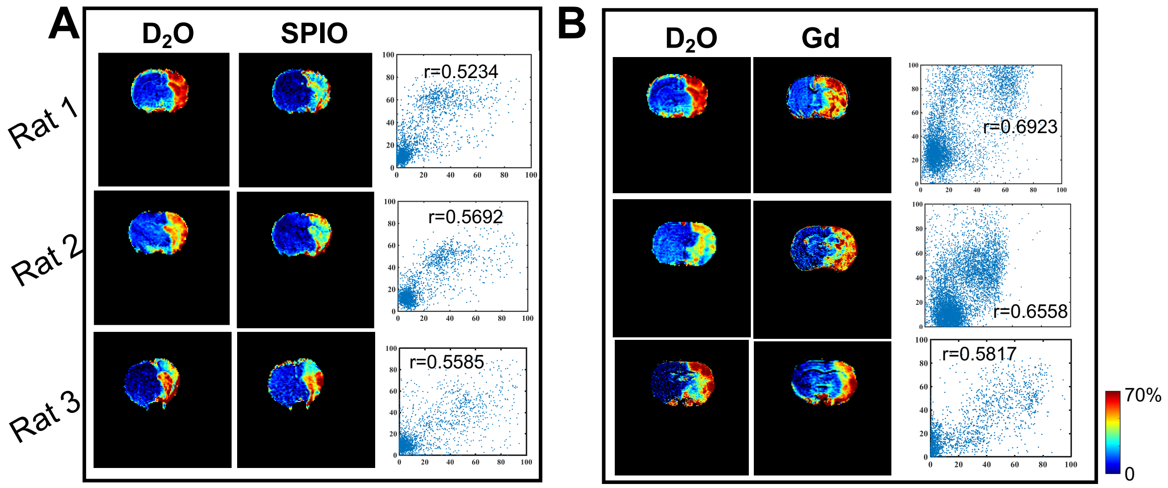

Figure S2. Comparison of the contrast maps between D_2O- and SPIO-MRI (A) and those between D_2O- and Gd -MRI (B) in all three animals.

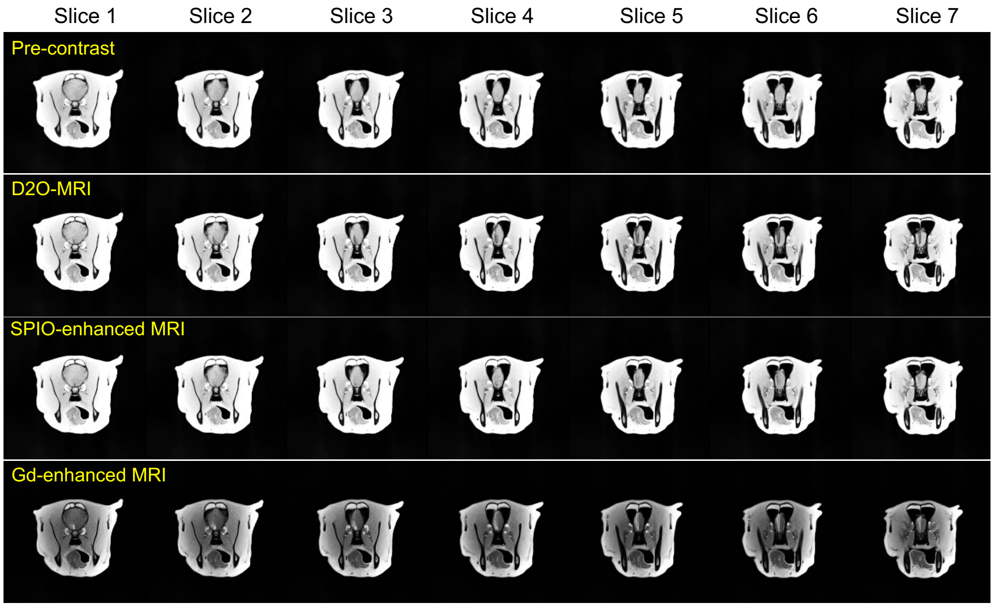

Figure S3. Images showing the dog brain pre- and post- SPIO or D_2O contrast before the injection of mannitol, and post- Gd contrast after the injection of mannitol.

Reference:

1. Lin AL, Qin Q, Zhao X, Duong TQ. Blood longitudinal (T1) and transverse (T2) relaxation time constants at 11.7 Tesla. MAGMA. 2012; 25: 245-9.
2. Stanisz GJ, Odrobina EE, Pun J, Escaravage M, Graham SJ, Bronskill MJ, et al. T1, T2 relaxation and magnetization transfer in tissue at 3T. Magn Reson Med. 2005; 54: 507-12.
3. de Graaf RA, Brown PB, McIntyre S, Nixon TW, Behar KL, Rothman DL. High magnetic field water and metabolite proton T1 and T2 relaxation in rat brain in vivo. Magn Reson Med. 2006; 56: 386-94.
